# Method of Obtaining Perfect Metallic Dies for Gold or Aluminum Swaged Plates, Restoring All Undercuts as on Plaster Model

**Published:** 1900-03

**Authors:** H. F. Grantvedt

**Affiliations:** Austin, Ill.


ARTICLE IV.

### METHOD OF OBTAINING PERFECT METALLIC DIES FOR GOLD OR ALUMINUM SWAGED PLATES, RESTORING ALL UNDERCUTS AS ON PLASTER MODEL.

H. F. GRANTVEDT, D. D. S., AUSTIN, ILL.

In reading over an article in November Items of Interest, by Dr. Oliver P. Wolfe, of Canton, Mass., under heading "Zinc Dies," a method which I have used several times occurred to me, and though somewhat like Dr. Wolfe's method, it yet differs.

When I have a patient desiring a metal swaged plate, and the mouth presents deep undercuts and large heels (such as a plaster impression will not come away from intact) I proceed as follows:

Select an impression cup as good a fit as possible, build up the center of the cup in inverted V form with wax. Try in the mouth so as not to get it too high or it will not allow the cup to be pressed up firmly when filled with plaster. Then oil the cup and wax, mix plaster about medium and smooth, carry to the mouth and press up firmly, allowing a fulness of plaster between the alveolus, cheeks and lips, letting it harden as much as possible before removing. Remove the cup, leaving the plaster in the mouth, which should be quite thin if done correctly. Now cut slightly in plaster over cuspid region, and from right to left side on under side of plaster. The anterior portion should now come away intact. Then place thumb against side portions, and same can usually be removed in two pieces, separating in middle line corresponding to the wax previously built on cup, and which, if smooth, would still be attached to the cup and not to the plaster in the mouth.

Place the parts of impression in cup, using hard wax to hold them secure; apply coating of shellac and wait till perfectly hard; then coat with sandarach varnish and allow it to dry thoroughly. Pour model, and have it harden thoroughly before separating, say twelve to twenty-four hours; too hasty separating may result in a change of shape, as plaster is supposed to expand one-five-hundredth of its volume. When perfectly dry, shellac and sandarach, same as was done with impression.

Now you will have exactly the size of the mouth, as two coats of varnish on impression reduces model, while two coats of varnish on model will give exact size as though made in an unvarnished impression, but being very much smoother. Then dry hard.

Place on clean glass slab, surface up, with a sufficient matrix around it (about five or six inches in diameter). Mix together plaster, fiber asbestos and powdered pumice, common sand or marble dust, in about these proportions: Asbestos, three parts; plaster two parts; and pumice or sand, one part. Mix in a large plaster bowl, water first, one-half to two-thirds full, adding slowly alternately of each according to their proportions until nearly all the water is taken up and the surface of the mass presents a milk-like color and consistency with no bubbles. Now mix thoroughly and pour into matrix, tapping slightly to insure settling. Let it stand over night. Then remove from matrix and trim; also cut a slight groove or ditch around the model, and you can pry it out of the mould or matrix formed by the mass.

Place this mould, firmly wired, on gas stove to dry out. When dry, turn on gas full pressure and bring the mass to a red heat, into which pour your molten metal, zinc or babbitt, and gradually turn off the gas until metal begins to crystallize; finally, say about one-half hour after pouring, turn off gas entirely, allowing to stand on stove until perfectly cool, when the metallic die can be removed and the counter made by first smoking die, then filling in undercuts with moldine or core and proceeding as usual. For an accurate counter, make sectionally.

By the above method it is possible to spring the plaster model out of the matrix, which is held together by the asbestos fiber, and therefore no cracking. You will also get a much smoother and more accurate die than you ever can in sand for such a case.—*Items of Interest.*

